# Comparative analysis of some bioactive compounds in leaves of different Aloe species

**DOI:** 10.1186/s13065-020-00720-3

**Published:** 2020-10-31

**Authors:** Bunea Andrea, Rugină Dumitrița, Copaciu Florina, Dulf Francisc, Veres Anastasia, Sonia Socaci, Pintea Adela

**Affiliations:** 1 Faculty of Animal Science and Biotechnologies, University of Agricultural Sciences and Veterinary Medicine, Calea Manastur, No. 3-5, 400372 Cluj-Napoca, Romania; 2 Faculty of Veterinary Medicine, University of Agricultural Sciences and Veterinary Medicine, Calea Manastur, No. 3-5, 400372 Cluj-Napoca, Romania; 3 Faculty of Agriculture, University of Agricultural Sciences and Veterinary Medicine, Calea Manastur, No. 3-5, 400372 Cluj-Napoca, Romania; 4 Faculty of Food Science, University of Agricultural Sciences and Veterinary Medicine, Calea Manastur, No. 3-5, 400372 Cluj-Napoca, Romania

**Keywords:** *Aloe* sp., Antioxidant activity, Ascorbic acid, Carotenoids, Fatty acids, HPLC, GC–MS

## Abstract

Although a vast number of *Aloe* species are known, only the *Aloe vera* and *Aloe arborescens* species are currently used by cosmetic and pharmaceutical industries. Therefore, the current study aims to complete the existent literature data with new information on the phytochemical composition of some lesser-known *Aloe* species, with the main focus on carotenoids and fatty acids. Among the analyzed species, *Aloe aculeata* and *Aloe ferox* had the highest content in carotenoids, the major pigments being lutein and β-carotene (according to HPLC analysis). The fatty acid profile of each *Aloe* species was analysed by GC–MS. Linolenic and linoleic acids were the major polyunsaturated fatty acids found in higher percent in *Aloe ferox*, *Aloe spectabilis* and *Aloe marlothii*. Instead, *Aloe aculeata* proved to have a distinct fatty acid profile, rich in monounsaturated fatty acids. Species such as *Aloe arborescens* and *Aloe marlothii* proved to have the highest antioxidant potential according to data of DPPH, ORAC, HPS assays, even if the richest one in vitamin C was found to be *Aloe spectabilis.* Though the scientific research is mainly focused on the common species *Aloe barbadensis*, the current data suggests that other *Aloe* species could receive more attention from industry part, being great sources of bioactive compounds.
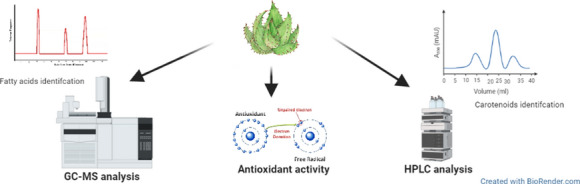

## Introduction

The genus *Aloe* (Family: Xanthorrhoeaceae) comprises over 500 species, of all the most well-known being *Aloe vera*. *Aloe* species, besides their widespread use as a food ingredient, have been intensely exploited in the traditional medicine of other cultures for their curative and therapeutic properties [1, 2].

The biological properties of *Aloe* such as anti-inflammatory, antimicrobial, anti-tumoral, and antioxidant, as well as wound healing ones are sustained by numerous in vitro and in vivo studies [1, 3]. These properties could be attributed to various compounds of the phytochemical profile of *Aloe* extracts, rather than to one single class of compounds. Focusing on the phytochemical content of leaves, in *Aloe vera* valuable molecules such as fatty acids (palmitoleic acid, linoleic acid), phenolic acids (phenol, vanillic, homovanillic, protocatechuic) and sterols (cholestanol) were found [4]. In a recent study, the phytochemical profile of the leaf exudates reported that many *Aloe* species contain free and glycosylated chromones, aloin and hydroxyaloins. Among the examined species in the same study, *A. marlothii*, and *A. melanacantha* were found to be the richest ones in total polyphenols, flavonoids and flavonols [1].

Fatty acids are bioactive compounds and an important part of the phytochemical content of *Aloe*, being widely used as feedstocks in the food industry and in the manufacture of soaps, detergents and cosmetics [23]. Nevertheless, both saturated and unsaturated fatty acids are relevant for their biological functions [24]. Polyunsaturated fatty acids (PUFAs), especially omega-3 and omega-6 fatty acids, are important dietary fats having numerous health benefits in humans. A right balance of these fatty acids in the human diet is crucial to prevent chronic diseases such as diabetes, obesity, cancer, and cardiovascular disease [25].

Carotenoids are molecules that exists in *Aloe* leaves, being components of the photosynthetic apparatus. They are known for their photoprotective and antioxidant properties. They play important roles for human health: as pro-vitamin A molecules (e.g. β-carotene), in eye protection (lutein and zeaxanthin), as antioxidants (lycopene, astaxanthin), for improvement of the cardiovascular health or cognitive functions [5].

Vitamin C (ascorbic acid) is a powerful dietary antioxidant, a water-soluble vitamin known to be essential to prevent scurvy or having positive effects in cardiovascular diseases [6] or diabetes [7]. It is also proved that it is able to improve the poor iron status and increase the absorption of iron [8]. Food and Drug Administration, FDA recommended that the amount of vitamin C for human consumption to be 75 mg per day for women and 90 mg for men. Phytochemicals in the *Aloes* may protect ascorbate from degradation in vivo [9]. From all known 500 species, *Aloe barbadensis* also known as *A. vera*, respective *A. arborescens* and *A. ferox* have the highest commercial importance [10]. The best-known and the most studied one *A. barbadensis* proved also its clinical effectiveness [11]. *A. arborescens* and *A. ferox* properties and their potential use as skin-conditioning agents or food additives were proved and accurately revised in few previously published papers [12–15]. Apart from these three *Aloe* species, little research has been conducted on the commercial potential of the other ones, although many of them are endemic in southern Africa and constitute an important component of the local flora from ethnobotanical, ecological, and social perspectives [2, 16]. Only *A. marlothii* is used in ethnoveterinary medicine for wound healing and reduction of infection and pain [17].

The phytochemical composition of different parts of *Aloe* might vary due to climate change [18], water stress, growth period and seasonality [19, 20], light intensity [21] as well as processing techniques, such as drying procedures [22].

**Fig. 1:**
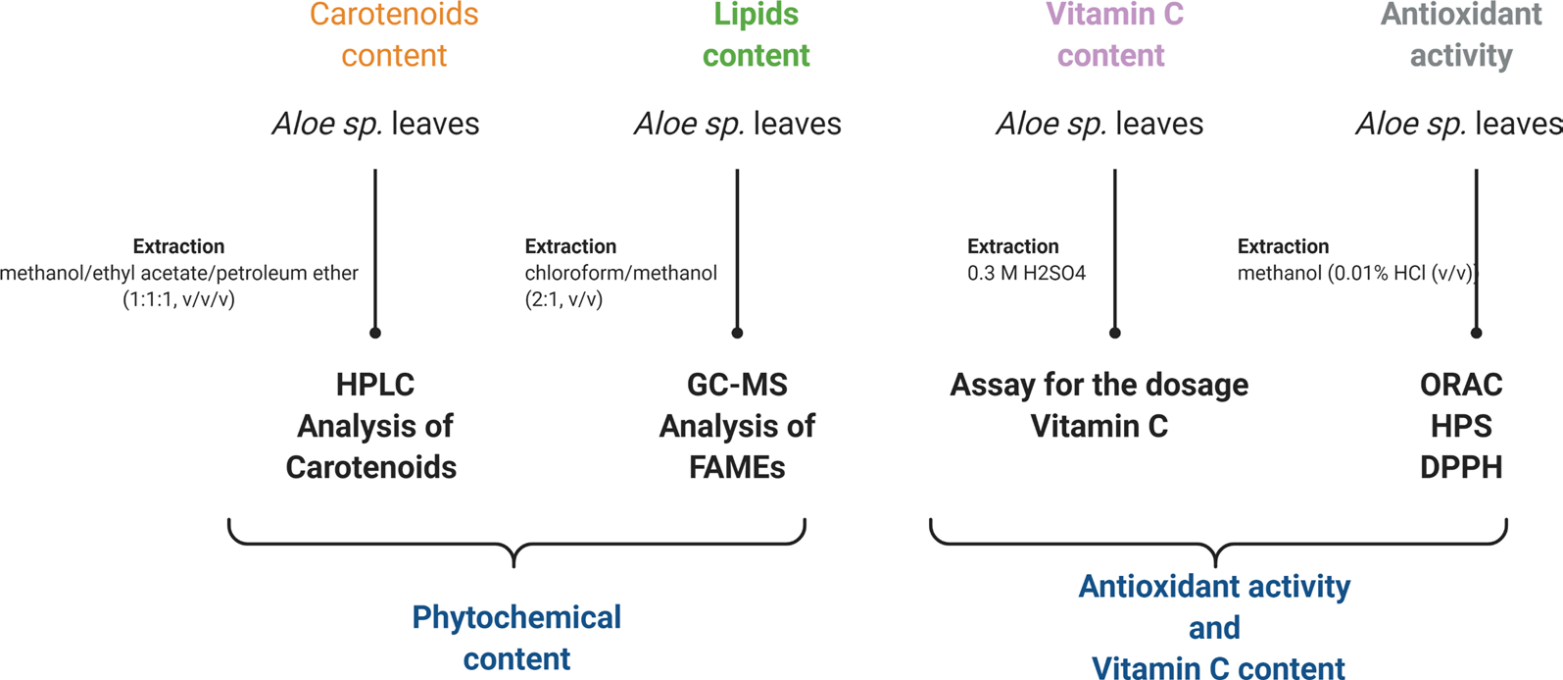
The representative flowchart of the experimental procedure

Regarding the phytochemical content of *Aloe*, the existent literature data is mostly focused on leaf gel [13, 23] and fewer on flower extracts [24]. However, as far as we know little work has been done on total leaf extracts. In this context, our study came to improve the existing knowledge on the phytochemical content of seven *Aloe* species. Therefore, we report here data about the carotenoids, fatty acids and vitamin C contents in whole leaf extracts of *A. aculeata*, *A. africana*, *A. arborescens*, *A. barbadensis*, *A. ferox*, *A. marlothii*, and *A. spectabilis*, as well as and their potential antioxidant activity (Fig. 1). To the best of our knowledge, this is the first study which offers a comprehensive overview about the carotenoids and fatty acids profile of some little investigated *Aloe* species, and certainly their individual phytochemical profile could be useful in varied industries.

## Materials and methods

### Chemicals

Methanol, ethyl acetate, petroleum ether, diethyl ether, tert-butyl methyl ether (TBME), sodium chloride, anhydrous sodium sulphate and chloroform, all were purchased from Merck (Darmstadt, DE). Lutein, zeaxanthin and β-carotene standards were provided by LGC Standards GmbH (Wesel, DE). 2,2′-Azobis(2-amidinopropane) dihydrochloride (AAPH), and 2,2-diphenyl-1-picrylhydrazyl (DPPH), 6-hydroxy-2,5,7,8-tetramethylchroman-2-carboxylic acid (Trolox) and the chemicals used for the lipid extraction and fatty acid methyl esters (FAMEs) preparation were all bought from Sigma Aldrich (St. Louis, MO, USA). The FAMEs standard (37-component FAMEMix) was purchased from Supelco (Bellefonte, PA, USA).

Seven different *Aloe* species: *A. aculeata*, *A. africana*, *A. arborescens*, *A. barbadensis*, *A. ferox* and *A. spectabilis*, were kindly supplied by Botanical Garden (Jibou, RO) (Coordinates 48° 51′ 12.28″ 2° 20′ 55.68″). The leaves of each species were collected and homogenized with a high-power homogeniser (MICCRA D-9, Müllheim, DE), in an ice bath, and then stored to − 20 °C before starting the extraction procedure.

### Extraction of carotenoids

Carotenoids were extracted from 5 g *Aloe* leaves using a previously described procedure [25]. After the *Aloe’s* leaves were minced and homogenised, carotenoids were extracted with a mixture of methanol/ethyl acetate/petroleum ether (1:1:1, v/v/v). This extraction procedure was repeated three times with the above-mentioned solvent mixture, until the residue became colourless. Then, the extracts of each repetition were mixed, and then the obtained final extract was supposed to a partition with water, diethyl ether and a saline solution. The upper phase formed was collected and evaporated to dryness, and stored at − 80 °C. Prior to the HPLC analysis, the residue was diluted with TBME and filtered (PTFE membrane filters, 0.45 μm).

### HPLC analysis of carotenoids

Analyses were performed on a Shimadzu HPLC system equipped with a binary pump delivery system LC-20 AT (Prominence), a degasser DGU-20 A3 (Prominence), diode-array SPD-M20 A UV–VIS detector. The column used was YMC C30 (24 cm × 4.6 mm; particle size 5 μm). The mobile phase was composed of solvent A: methanol/TBME/water (81:15:4, v/v/v) and solvent B: TBME/methanol/water (90:7:3, v/v/v). The gradient started with 1% B at 0 min and increased to 100% B at 160 min according to the method described previously [26]. The flow rate was adjusted to 0.8 mL/min. The DAD operated in the range of 300–600 nm for the acquisition of UV–Vis spectra and the chromatograms were extracted at 450 nm. Individual carotenoids were identified by comparing their retention time, UV–Vis spectra (ʎ_max_, spectral fine structure) with those of the available standards and literature data. The quantitative analysis of carotenoids was based on external calibration using β-carotene (R^2^ = 0.9912) and lutein (R^2^ = 0.9996) standard solutions, in the range 1–100 μg/ml.

### Lipid extraction

Total lipids were extracted using the classical method described by Folch et al. [27]. Briefly, the extraction was done using a chloroform/methanol mixture. The extraction procedure started with the homogenization of *Aloe* leaves (5 g) in methanol using a high-power homogeniser (MICCRA D-9, Müllheim, DE). Then, chloroform was added and the homogenisation procedure continued for another 2 min. The mixture was filtered and the solid residue was suspended in chloroform/methanol (2:1, v/v) and homogenised again for 3 min. After filtration, the residue was further washed with chloroform/methanol (2:1, v/v). The filtrates and washes were combined, and then all were washed with 0.88% aqueous potassium chloride followed by methanol/water (1:1, v/v) solution. The purified lipidic layer (bottom) was filtered, dried over anhydrous sodium sulphate, and then evaporated to dry in a rotary evaporator. Total lipids were determined gravimetrically and stored in chloroform at − 20 °C until were analysed. The recovered oils were transferred to vials with 2 ml chloroform (stock solution) and stored at − 18 °C until further analysis.

### GC–MS analysis of FAMEs

The fatty acids profile of *Aloe* species was determined by gas chromatography coupled with mass-spectrometry. Fatty acids were analysed in their methyl ester form. FAMEs were prepared from total lipid extracts using the acid-catalysed transesterification procedure described previously [28]. The esters were extracted twice with hexane; the combined extracts were dried over anhydrous sodium sulphate and filtered. For FAMEs analysis, a Perkin-Elmer Clarus 600T GC–MS was used. The apparatus was equipped with a Supelcowax 10, 60 m × 0.25 mm i.d., 0.25-m film thickness (Supelco Inc., Bellefonte, PA, USA) capillary column. The injector temperature was set at 210 °C. The oven temperature began at 140 °C, then it was increased to 220 °C by 7 °C/min and was maintained at 220 °C for 23 min. The flow rate of the carrier gas He and the split ratio were 0.8 ml/min and 1:24, respectively. The ionisation energy for the positive ion electron impact (EI) mass spectra was 70 eV with a trap current of 100 μA and a source temperature of 150 °C. Mass scans were performed within the range of m/z 22–395 at a rate of 0.14 scan/s with an intermediate time of 0.02 s between the scans. The injection volume was 0.5 μl. FAMEs were identified by comparison of their retention times with those of known standards (37-component FAME Mix, Supelco no. 47885-U) and with mass spectra obtained with compounds from our database (NIST MS Search 2.0). Each fatty acid was expressed as peak area percentage of total fatty acids.

### Vitamin C determination

The procedure to obtain the extracts and the protocol of the titrimetric method used for the vitamin C determination from the leaves of the selected *Aloe* species, was described previously [29]. Briefly, 30 g *Aloe* leaves were homogenised with 100 ml of 0.3 M H_2_SO_4_ and then the extract was filtrated. 10 ml of filtrated extract was mixed with 30 ml H_2_O, 5 ml KI 10%, 1 ml H_2_SO_4_ (0.3 M) and 10 ml KIO_3_ (0.01 M). The excess of iodine was titrated against 0.01 M sodium thiosulphate (NaS_2_O_3_). The amount of vitamin C was calculated as a difference of meq KIO3 and meq Na_2_S_2_O_3_.

### Antioxidant activity assays

For the antioxidant activity analysis, extracts were obtained starting from 5 g of *Aloe* leaves grinded with 40 mL acidified methanol (0.01% HCl (v/v)) by a homogenizer (Miccra D-9 KT Digitronic, Bergheim, Germany) and then was concentrated to 1 ml.

The oxygen radical absorbance capacity (ORAC) was measured and calculated as it was previously described [30]. Briefly, a fluorescein solution (8.16 × 10^−5^ mM) in phosphate buffer (75 mM, pH = 7.4) was incubated with 25 μl each standard (Trolox) or sample (*Aloe* extract) for 30 min, at 37 °C. Then, the reaction was initiated by adding 25 μl 2,2′-azobis-2-amidinopropane (AAPH, 153 mM in PBS) and the fluorescence was measured kinetically at excitation wavelength 485 nm and emission wavelength 535 nm, every minute using the fluorescence microplate reader BioTek (Synergy HT, BioTek Instruments, Winooski, VT). The reported ORAC values were calculated and expressed as μmol Trolox equivalents (TE) per liter for gram for solid sample according to ORAC protocol adapted on the microplate reader [30].

The hydrogen scavenging assay (HPS) was carried out following the procedure of Ruch et al. [31]. Briefly, a mixture of 3.4 ml phosphate buffer solution (1 M, pH = 7.4), 5 μl of *Aloe* extract or Trolox standard and 0.6 ml of H_2_O_2_ (40 mM in phosphate buffer 1 M with pH = 7.4) was prepared and its absorbance was read at 230 nm, against a blank solution (phosphate buffer without H_2_O_2_). The absorbance was read by a spectrophotometer (JASCO V-630 series, International Co., Ltd., Japan).

The potential to scavenge the 2,2-diphenyl-1-picrylhydrazyl (DPPH) radical was evaluated using the method described by Brand-Williams et al. [32]. Briefly, 250 μl of DPPH solution (80 μM in 95% methanol) was allowed to react 30 min in the dark with 35 μl *Aloe* extract and then the absorbance of each sample and standard was measured at 515 nm [34]. Absorbances were recorded with the microplate reader BioTek (Synergy HT, BioTek Instruments, Winooski, VT). All the values resulted from ORAC, HPS, DPPH assays were expressed in the same unit, as μmol TE/g FW, being calculated similar as in a previous study [33].

### Statistical analysis

PCA analysis. For the characterisation of the studied *Aloe* species, the data obtained from chromatographic and spectrophotometric analyses was subjected to principal component analysis (PCA) with cross-validation (full model size and centre data), using Unscrambler X version 10.5 software (CAMO Software AS, Oslo, Norway).

All extractions and chromatographic analyses were performed in triplicate. The results for HPLC, GC–MS analyses and antioxidant assays are presented in tables as the mean ± standard deviation. Significant differences between samples were analysed with one-way ANOVA post hoc tests and pairwise multiple comparisons were conducted using Tukey’s test. Significant differences were reported based on *P *< 0.05. Statistical analyses were performed using the SPSS Statistics 23.0.

## Results and discussion

### Carotenoid content in whole Aloe leaves extracts

**Fig. 2:**
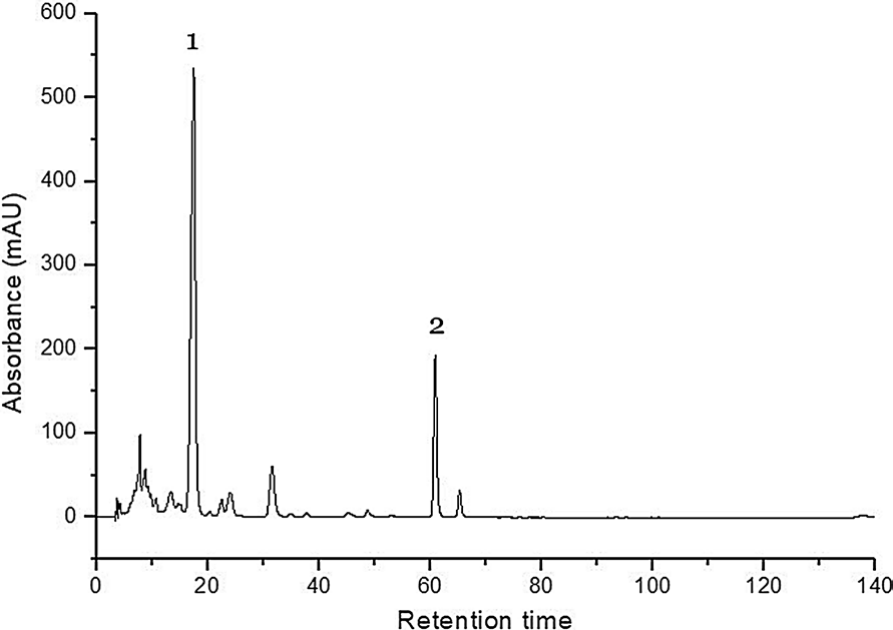
HPLC chromatogram of carotenoids in *Aloe aculeata* leaves. Peak 1- Lutein; Peak 2- β-carotene

Reversed Phase High Performance Liquid Chromatography (RP-HPLC) is the analytical technique preferred by researchers for the carotenoids separation, quantification and their structural characterization [34]. The specific spectral characteristics were used for individual carotenoid identification, especially when corroborated with the chromatographic behaviour. In all tested samples, *β*-carotene and lutein were identified (Fig. 2), as the predominant *β*-carotene and xanthophyll compounds, with a varied ratio among species (Table 1).

**Table 1:** Carotenoid and vitamin C composition of *Aloe* species

*Aloe* species	Carotenoids mg/kg FW			
	Lutein	β-Carotene	Total carotenoids	Vitamin C (mg/100 g FW)
*Aloe aculeata*	9.02 ± 0.61^a^	7.47 ± 0.41^a^	16.49 ± 1.20^a^	1.17 ± 0.03^c^
*Aloe africana*	6.60 ± 0.19^b^	3.56 ± 0.18^bc^	10.19 ± 0.27^c^	2.34 ± 0.16^b^
*Aloe arborescens*	5.64 ± 0.38^b^	4.12 ± 0.25^b^	9.77 ± 0.25^c^	2.34 ± 0.09^b^
*Aloe barbadensis*	4.00 ± 0.27^c^	2.76 ± 0.14^d^	6.75 ± 0.42^d^	1.16 ± 0.07^c^
*Aloe ferox*	9.00 ± 0.34^a^	4.06 ± 0.25^b^	13.06 ± 0.80^b^	2.34 ± 1.13^b^
*Aloe marlothii*	8.43 ± 0.38^a^	7.41 ± 0.37^a^	15.84 ± 0.52^a^	2.01 ± 0.09^b^
*Aloe spectabilis*	6.00 ± 0.26^b^	3.12 ± 0.13 ^cd^	9.12 ± 0.28^c^	5.85 ± 0.25^a^

*FW* fresh weight, *SD* standard deviation

Values followed by different letters within each column denote a significant difference and those followed by same letters denote no significant difference at P < 0.05

*A. barbadensis* leaves proved to have the lowest β-carotene content (2.76 mg/kg) among the other species taken into study, but this content seems to be significantly lower than that observed by Ozsoyet et al. (15.51 mg/kg) [35]. It has to be taken into account that growing location and some environmental factors could influence the carotenoid composition in *Aloe* species. In other species as *A. arborescesns* and *A ferox* the β-carotene concentrations detected were higher than ⁓ 4 mg/kg FW, but the species with the highest values for β-carotene concentrations were *A. aculeata* and *A. marlothii* of about 9 mg/kg FW.

A similar tendency as that noted for the β-carotene content in leaves extracts of *Aloe* species tested, was observed for the lutein concentration too, which varied within 4.00 mg/kg (for *A. barbadensis*) and 9.02 mg/kg (for *A. aculeata*) range. However, *Aloe aculeata and Aloe ferox* have the highest lutein content, which make these species remarkable as the richest ones in carotenoids.

### Fatty acid composition in whole Aloe leaves extracts

**Fig. 3:**
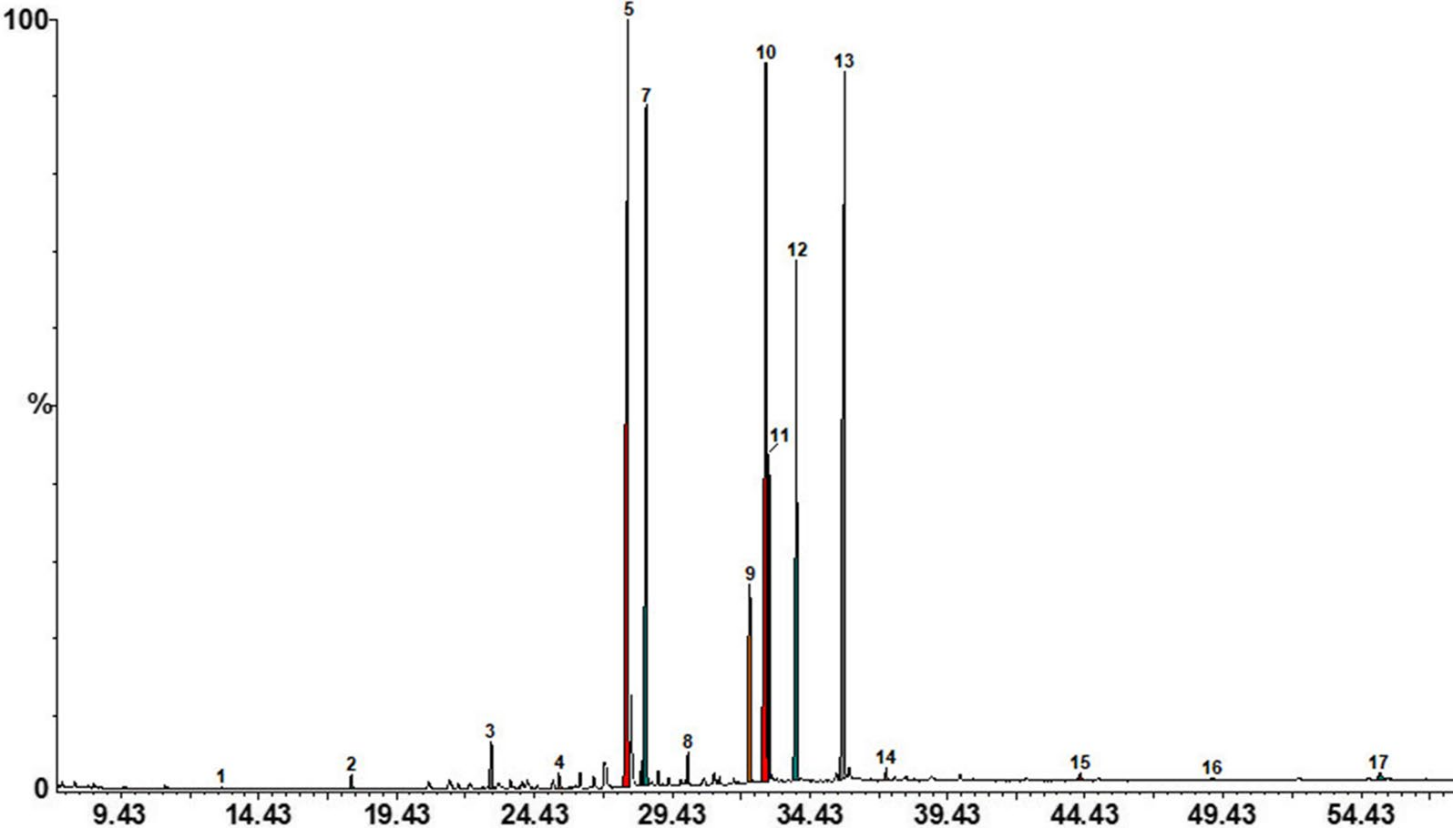
Characteristic GC–MS chromatogram of the fatty acids profile of *Aloe aculeata* leaves. The numbers written on chromatogram correspond to the identified fatty acids accordingly: 1-Capric acid (C10:0), 2-Lauric acid (C12:0), 3-Myristic acid (C14:0), 4-Pentadecyclic acid (C15:0), 5-Palmitic acid (C16:0), 6-Palmitoleic acid (C16;1, n − 9), 7-Cis-7 hexadecenoic acid (C16:1, n − 7), 8-Margaric acid (C17:0), 9-Stearic acid (C18:0), 10-Oleic acid (C18:1, n − 9), 11-Vaccenic acid (C18:1 n − 7), 12-Linoleic acid (C18:2 n − 6), 13-Linolenic acid (C18:3 n − 3), 14-Arachidonic acid (C20:0), 15-Behenic acid (C22:0), 16-Tricosylic acid (C23:0), 17-Lignoceric acid (C24:0)

The data for the total lipid contents of *Aloe* leaves, expressed on the basis of fresh weight, are summarized in Table 2. GC–MS analysis revealed the presence of 17 fatty acids, which can be found in leaves of each *Aloe* species analysed (Fig. 3 and Table 2). Saturated fatty acids (SFA) were identified in the range of C_10_–C_24_. One of the saturated fatty acid found in all leaves of *Aloe* species was palmitic acid (C16:0). As can be seen in the Table 2, *Aloe aculeata* and *Aloe barbadensis* possess in their leaves the highest percent of palmitic acid, of about 24.13, respective 26.48 comparing to other *Aloe* species. Another saturated fatty acid identified in samples was stearic acid (C18:0) in *Aloe africana*, respective *Aloe spectabilis*. The most prominent polyunsaturated fatty acids found in *Aloe* leaves were linoleic acid (C18:2 n − 6) and linolenic acid (C18:3 n − 3). In particular, linoleic acid (C18:2 n − 6) was found in higher percent in *Aloe spectabilis*, *Aloe arborescens* and *Aloe ferox* leaves, values ranged from 19.61 to 22.5 percent. Regarding linolenic acid (C18:3 n − 3), it is highly represented in all species apart of *Aloe aculeata,* in which it is less represented with about 15–20%. These two polyunsaturated fatty acids comprise about 50% of the fatty acids of *Aloe* leaves (Table 2).

**Table 2:** Fatty acid composition of total lipids extracted from *Aloe* species

No.	Fatty acid (%)*	*Aloe aculeata*	*Aloe africana*	*Aloe arborescens*	*Aloe barbadensis*	*Aloe ferox*	*Aloe marlothii*	*Aloe spectabilis*
1	Capric acid (C10:0)	0.04 ± 0.00^e^	0.17 ± 0.00^b^	0.19 ± 0.01^a^	0.09 ± 0.00^d^	0.02 ± 0.00^f^	0.08 ± 0.00^d^	0.12 ± 0.01^c^
2	Lauric acid (C12:0)	0.24 ± 0.01^f^	0.64 ± 0.03^b^	0.56 ± 0.02^c^	0.42 ± 0.02^d^	0.32 ± 0.01^e^	0.51 ± 0.02^c^	0.73 ± 0.03^a^
3	Myristic acid (C14:0)	0.83 ± 0.04^f^	2.54 ± 0.12^a^	2.09 ± 0.10^b^	1.01 ± 0.05^ef^	1.44 ± 0.07^d^	1.09 ± 0.05^e^	1.73 ± 0.08^c^
4	Pentadecyclic acid (C15:0)	0.26 ± 0.01^e^	0.54 ± 0.01^bc^	0.78 ± 0.05^a^	0.46 ± 0.02^d^	0.51 ± 0.04^bcd^	0.5 ± 0.02^cd^	0.58 ± 0.02^b^
5	Palmitic acid (C16:0)	24.13 ± 1.17^ab^	22.42 ± 1.11^bc^	21.70 ± 1.12^bc^	26.48 ± 1.38^a^	20.44 ± 0.99^c^	24.48 ± 1.23^ab^	20.65 ± 0.98^c^
6	Palmitoleic acid (C16:1 n − 9)	0.53 ± 0.02^d^	1.44 ± 0.07^b^	3.25 ± 1.15^a^	0.91 ± 0.04^c^	1.61 ± 0.81^b^	1.08 ± 0.05^c^	1.48 ± 0.72^b^
7	Cis-7 hexadecenoic acid (C16:1 n − 7)	14.16 ± 0.72^a^	0.48 ± 0.02^d^	0.37 ± 0.01^d^	2.81 ± 0.13^b^	0.89 ± 0.04^cd^	1.56 ± 0.08^c^	0.65 ± 0.03^d^
8	Margaric acid (C17:0)	0.54 ± 0.02^f^	1.14 ± 0.06^d^	0.83 ± 0.04^e^	1.93 ± 0.09^a^	0.85 ± 0.04^e^	1.73 ± 0.08^b^	1.52 ± 0.07^c^
9	Stearic acid (C18:0)	4.61 ± 0.23^c^	5.72 ± 0.28^a^	5.35 ± 0.27^ab^	4.69 ± 0.22^bc^	4.74 ± 0.23^bc^	4.39 ± 0.22^c^	5.85 ± 0.28^a^
10	Oleic acid (C18:1 n − 9)	18.46 ± 0.94^a^	7.03 ± 0.34^b^	7.08 ± 0.33^b^	7.73 ± 0.39^b^	7.26 ± 0.34^b^	7.53 ± 0.37^b^	7.00 ± 0.35^b^
11	Vaccenic acid (C18:1 n − 7)	5.03 ± 0.24^a^	1.34 ± 0.06^b^	0.55 ± 0.02^e^	1.23 ± 0.06^bc^	0.89 ± 0.04^d^	1.03 ± 0.05^cd^	0.98 ± 0.05^cd^
12	Linoleic acid (C18:2 n − 6)	10.75 ± 0.54^e^	17.37 ± 1.85 ^cd^	19.92 ± 0.62^ab^	15.67 ± 0.81^d^	19.61 ± 1.00^bc^	17.77 ± 0.93^bcd^	22.25 ± 1.07^a^
13	Linolenic acid (C18:3 n − 3)	19.50 ± 0.97^c^	36.43 ± 1.81^ab^	34.74 ± 1.72^b^	36.94 ± 1.79^ab^	40.55 ± 2.03^a^	37.89 ± 1.95^ab^	33.55 ± 1.68^b^
14	Arachidonic acid (C20:0)	0.29 ± 0.01^c^	0.57 ± 0.02^b^	0.77 ± 0.04^a^	0.73 ± 0.03^a^	0.23 ± 0.01^c^	0.71 ± 0.03^a^	0.69 ± 0.03^a^
15	Behenic acid (C22:0)	0.20 ± 0.01^d^	0.65 ± 0.03^c^	1.02 ± 0.04^a^	0.69 ± 0.03^bc^	0.22 ± 0.01 ^cd^	0.73 ± 0.03^bc^	0.76 ± 0.03^b^
16	Tricosylic acid (C23:0)	0.09 ± 0.00^e^	0.51 ± 0.02^a^	0.22 ± 0.01^c^	0.41 ± 0.02^b^	0.16 ± 0.01^d^	0.38 ± 0.02^b^	0.52 ± 0.02^a^
17	Lignoceric acid (C24:0)	0.33 ± 0.02^d^	1.02 ± 0.05^a^	0.60 ± 0.03^c^	0.80 ± 0.03^b^	0.26 ± 0.01^d^	0.75 ± 0.03^b^	0.95 ± 0.05^a^
	∑SFAs	31.57 ± 1.63^ab^	35.91 ± 1.75^a^	34.09 ± 1.63^a^	34.72 ± 1.70^a^	29.21 ± 1.39^b^	35.35 ± 1.76^a^	34.10 ± 1.68^a^
	∑MUFAs	38.18 ± 1.93^a^	10.29 ± 0.52^bc^	11.26 ± 0.55^bc^	12.67 ± 0.62^b^	10.64 ± 0.55^bc^	11.20 ± 0.56^bc^	10.10 ± 0.49^c^
	∑PUFAs	30.25 ± 1.58^c^	53.80 ± 2.68^ab^	54.65 ± 2.73^ab^	52.61 ± 2.52^b^	60.15 ± 3.15^a^	55.69 ± 2.68^ab^	55.80 ± 2.72^ab^
	PUFAs/SFAs	0.96- ± 0.04^c^	1.50 ± 0.08^b^	1.60 ± 0.08^b^	1.52 ± 0.08^b^	2.06 ± 0.10^a^	1.52 ± 0.08^b^	1.64 ± 0.09^b^
	n − 6/n − 3 PUFAs	0.55 ± 0.02^bc^	0.48 ± 0.02 ^cd^	0.57 ± 0.03^b^	0.42 ± 0.04^d^	0.48 ± 0.02 ^cd^	1.64 ± 0.09^b^	0.66 ± 0.03^a^
	Total lipid content (g/100 g FW)	2.768 ± 0.32^d^	3.086 ± 0.81^c^	3.121 ± 0.82^c^	3.173 ± 0.56^c^	4.105 ± 0.49^b^	3.995 ± 0.37^b^	4.323 ± 0.93^a^

* % of total fatty acids; values are expressed as mean ± standard deviation. Values followed by different letters within each column denote a significant difference and those followed by same letters denote no significant difference at P < 0.05

Cis-7 hexadecenoic acid (C16:1 n − 7) and oleic acid (C18:1 n − 9) were better represented in *Aloe aculeata* leaves compared to the other species analyzed. The content of oleic acid (18:1 n − 9) was quite similar with that of stearic acid (18:0) in all *Aloe* species, with average percentage of 7.28 (between 7.00 and 7.73) and 5.12 (4.39–5.85), respectively.

*Aloe aculeata* distinguishes itself from the other species having a different fatty acids profile, with palmitic acid (24.13%) and linolenic acid (19.50%) as the major fatty acids. Interesting is the fact that also oleic acid (C18:1 n − 9, 18.46%) and palmitoleic acid (16:1 n − 7, 14.16%) were higher represented in *A. aculeata*, comparing to other species.

Traces of capric acid (C10:0), lauric acid (C12:0), myristic acid (14:0), pentadecanoic acid (C15:0), margaric acid (C17:0), arachidonic acid (C20:0), behenic acid (C22:0), trycosilic acid (C23:0), lignoceric acid (C24:0) were found in all species of *Aloe* leaves taken into study. It is also interesting to note the relatively high percentage of the very long chain fatty acids (> 20 °C) in *A. africana*, *A. arborescens*, *A.barbadensis*, *A.marlothii* and *A. spectabilis*.

Regarding the saturated fatty acid (SFA) representation, significant differences (P < 0.05) between the species can be seen (Table 2). *Aloe ferox* presented the highest PUFAs percentage 60.15 and the lowest in SFAs 29.20 compared to the other species, all differences being statistically significant. A significant PUFAs, monounsaturated fatty acids (MUFAs), and SFAs percentage were seen in *A. africana*, *A. arborescens*, *A. barbadensis*, *A. marlothii*, and *A. spectabilis*, with no significant variations between these species, the medium values being 54.51, 34.83, and 11.10, respectively. The PUFAs/SFAs ratios were ≥ 1.50 in six out of seven *Aloe* species, and among all *A. ferox* ratio being significantly higher. Overall, no major differences were observed in terms of the n-6/n-3 PUFAs ratio. Interestingly, *A. aculeata* presented a more balanced composition concerning the type of fatty acid. MUFAs had the highest proportion (38.18%), significantly higher than the other species, followed by SFAs (31.57%) and PUFAs (30.25%).

As far as we known, there are no data available regarding the fatty acid composition of *Aloe* leaves, only few studies were found about fatty acids existence in gel leaves of *Aloe*. But, in the leaf gel of *Aloe ferox* was found that the major fatty acid is linoleic acid, which represent a value ~ twofold and ~ 68-fold higher than that for palmitic acid and α-linolenic acid [13]. In contrast, in the leaf gel of *Aloe barbadensis* the major fatty acid found was linoleic acid [4], whereas in the flowers of *Aloe barbadensis* species the representative fatty acids are myristoleic acid (C14:1 n − 9, 31.2%) and palmitic acid (C16:0, 22.86%) [36]. The total lipid analysis of eight *Aloe* gels (including from *Aloe barbadensis*, *Aloe arborescens* and *Aloe ferox*) revealed a difference among species regarding the concentration of fatty acids [37]. *Aloe arborescens* contains significantly higher concentration of fatty acids than *Aloe ferox* or *Aloe barbadensis*, which means that a higher concentration of fatty acids indicate the existence of an efficient coat of the plant working as a barrier toward stress factors [38].

There are several studies which sustain that low values of the dietary n-6/n-3 essential fatty acids (ranging from 1 to 5) and PUFAs/SFAs (ranging from 1 to 1.5) ratios can reduce significantly the risk of cardiovascular disease and cancer [16]. Moreover, among polyunsaturated fatty acids (PUFAs), linoleic acid has been shown to be the most potent fatty acid for lowering the plasma triacylglycerols, low-density and high-density lipoprotein cholesterol [17].

### Vitamin C content

*Aloe spectabilis* compared with all other species showed the highest content in vitamin C, while other species like *A. africana*, *A. arborescens*, and *A. ferox* presented all similar values (Table 1). Vega-Gálvez et al. in a study done to observe the effects of high pressures (500 MPa) on vitamin C content from *Aloe vera* gel, measured an initial content of 126.37 mg/100 g dry weight [39].

### PCA analysis

**Fig. 4:**
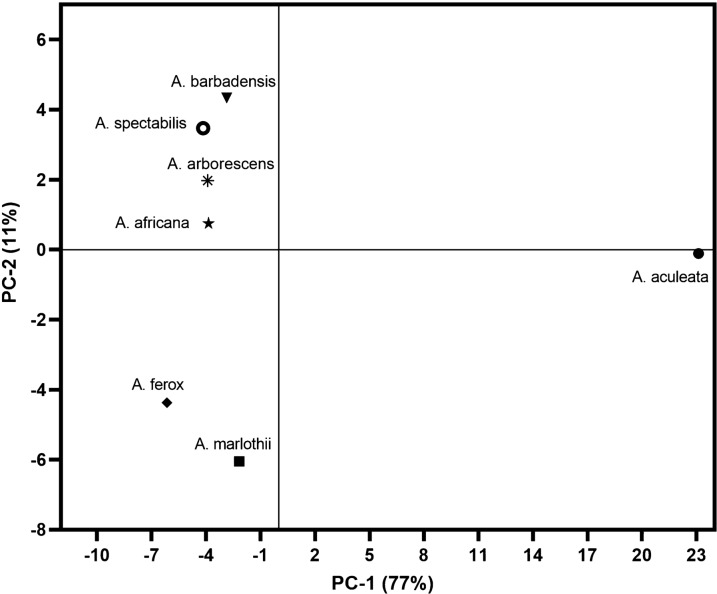
Principal components analysis bi-plots of 7 *Aloe* species based on their fatty acids profiles, carotenoids and vitamin C content. The first two components together explained 88% of the data variation

The data resulted from carotenoids, fatty acids and vitamin C analyses were subjected to the Principal Component Analysis (PCA) in order to underline the samples’ similarities and differences based on their specific chemical composition. In this regard a matrix containing the 7 *Aloe vera* samples and 21 variables for each sample (including concentration values for 17 fatty acids, lutein, β-carotene, vitamin C and total amount of carotenoids) was computed. The calculation was performed using a mean center data model (which allows to subtract the column means from every variable before analysis), with cross validation and single value decomposition algorithm. Thus, using this chemometric method, the two principal components explained 88% of the overall variance (77% and 11% for PC-1 and PC-2, respectively) dividing the studied samples into 3 distinct clusters (Fig. 4). The first cluster included *A. barbadensis*, *A. spectabilis*, *A. arborescens* and *A. africana*, the second *A. ferox* and *A. marlothii* and the third one is represented by *A. aculeata*, which has a very distinctive pattern compared to other *Aloe* samples.

**Fig. 5:**
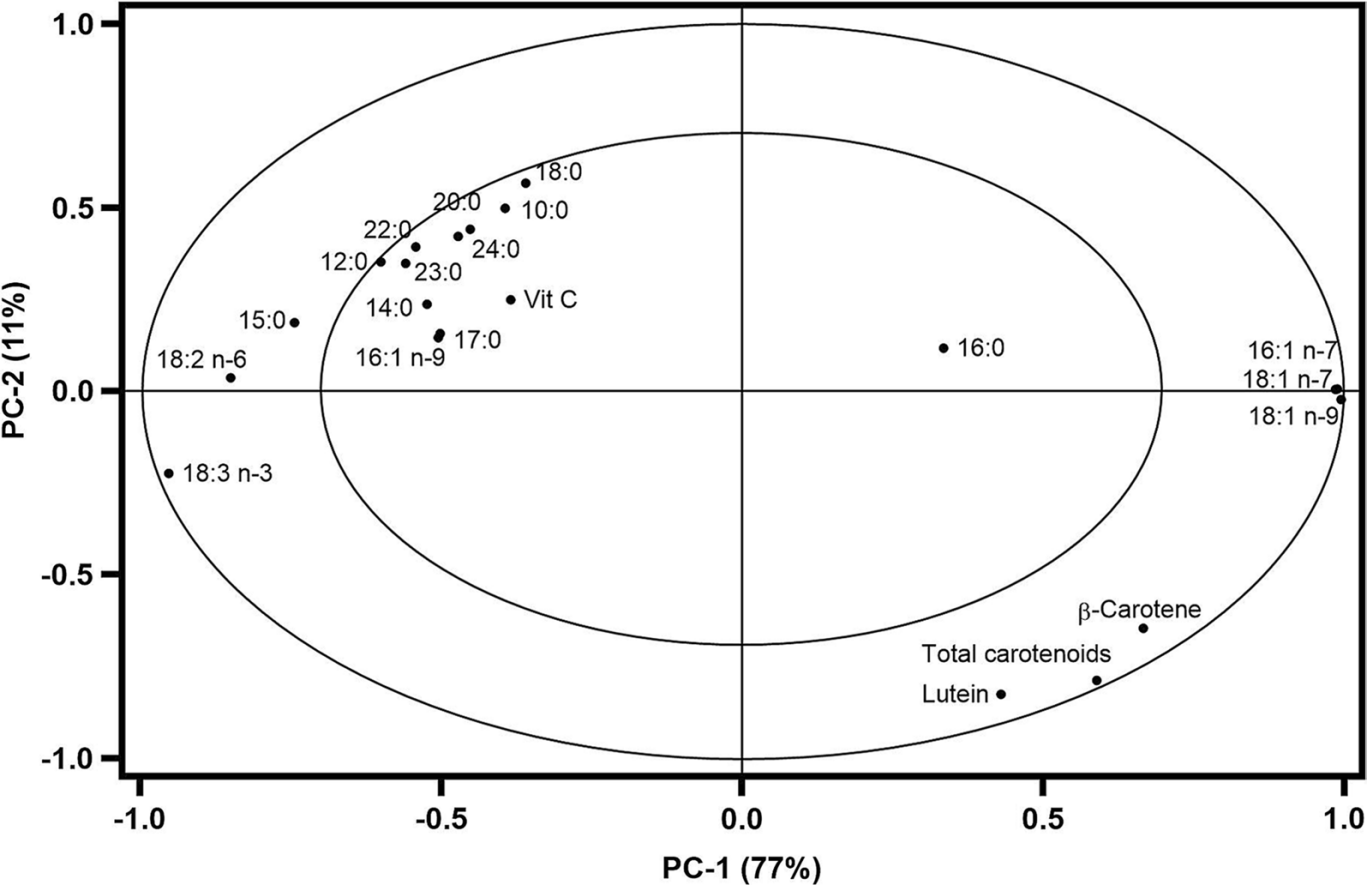
Correlation loadings bi-plots for the *Aloe* samples

The correlation loadings bi-plot was also computed in order to point out the correlations between *Aloe* species and determined bioactive compounds (Fig. 5). The compounds within the inner ellipse indicate 50% of explained variance, while the outer ellipse indicates 100% of explained variance. In this way, the importance of individual variables is visualized more clearly. Thus, in the case of *A. aculeata*, the correlation loading bi-plot (Fig. 5) highlighted three marker compounds, namely: C18:1n − 7, C18:1n − 9 and C16:1n − 7 fatty acids.

### Antioxidant activity

**Fig. 6:**
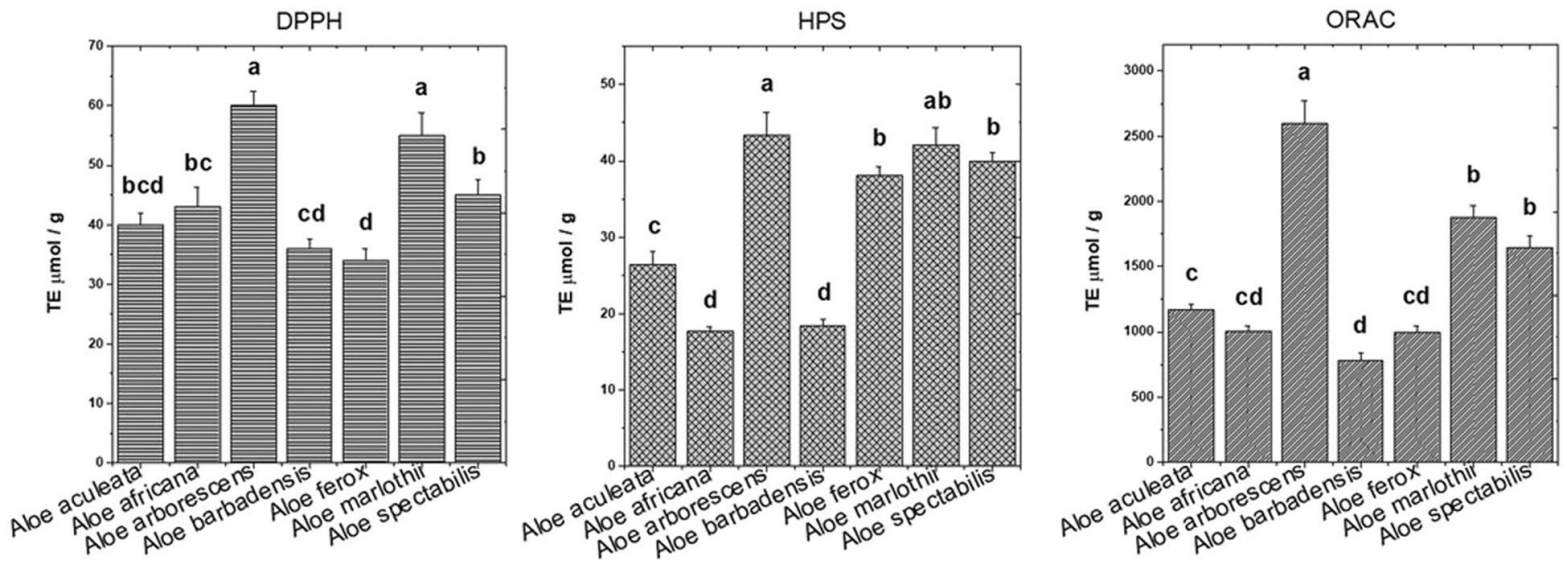
Antioxidant activities of the whole leaf extracts of *Aloe* species, resulted according to DPPH, ORAC, HPS radical scavenging assays. Values followed by different letters within each column denote a significant difference and those followed by same letters denote no significant difference at P < 0.05. Error bars represent standard deviation (SD) from three independent experiments

Since antioxidant molecules (e.g., ascorbate, carotenoids) could add an extra value in pharmaceutical products for instance, we evaluated the potential antioxidant activity for the *Aloe* leaves extracts. Three different assays such as DPPH, ORAC, and HPS were used to evaluate the free radical scavenging potential of the *Aloe* leaves extracts (Fig. 6). The scavenging potential against peroxyl radical of *Aloe* species was assessed by ORAC assay revealed that the highest ORAC values were obtained for *Aloe arborescens* and *Aloe marlothii*. Regarding the HPS assay assessment, *Aloe arborescens* and *Aloe marlothii*, followed by *Aloe ferox* and *Aloe spectabilis* proved to have the highest potential to scavenge hydrogen peroxide, known as toxic by-product of the oxygen metabolism in viable cells.

Based on three different assays, *A. arborescens* proved to exert the highest scavenging activity (60 µmol TE/g FW for DPPH assay, 2600 µmol TE/g FW for ORAC assay, and 43.35 µmol TE/g FW for HPS assay) followed by *A. marlothii* (55 µmol TE/g FW, 1876 µmol TE/g FW, and 42.00 µmol TE/g FW). Certainly, both carotenoids and vitamin C contributed to the antioxidant activity of *A. arborescens* extract. However, there are other compounds with antioxidant potential in the extracts, since *Aloe spectabilis* and *Aloe ferox* also have a high carotenoid and vitamin C content, but had a lower antioxidant activity than *Aloe arborescens*.

Our observations seem to be consistent with those available in scientific literature. For example, in one study the phytochemical profile and the antioxidant activity of different leaf portions of *A. arborescens* and *A. barbadensis* were compared. It was found that the ORAC radical scavenging potential was higher for *A. arborescens* than for *A. barbadensis*, while the DPPH assay indicated a reversed order, without a statistic significance [3]. When ORAC values of seven *Aloe* species were compared, the highest value was reported for *A. arborescens* (2135.1 µmol TE/100 g FW), which was significantly higher than that for *A. ferox* (525.72 µmol TE/100 g FW), and *A. barbadensis* (1234.4 µmol TE/100 g FW) [10]. In the study conducted by Cardarelli et al. the scavenging activity profiles obtained by DPPH and ORAC assays, followed the trend *A. marlothii *< *A. ferox *< *A.arborescens *< *A. barbadensis* [1]. Similar ORAC radical scavenging activities were obtained for *A. barbadensis* and *A. ferox*, when the lyophilized leaf gel and the ethanolic leaf extracts were compared [4, 13].

Here, from all analysed species, *A. arborescens*, *A. marlothii*, and *A. spectabilis* had a similar potential to scavenge the hydrogen peroxide by HPS assay, but among this species, *A. arborescens* had the highest scavenge potential (43.35 µmol TE/g FW). In contrast, a low scavenging activity was observed for *A. africana* (17.72 µmol TE/g FW) and *A. barbadensis* (18.43 µmol TE/g FW). In a recent study, it was proved that the agro-climatic conditions could affect phytochemicals, the Total Phenolic Content (TPC) and the antioxidant potential of *A. barbadensis.* Thus, the antioxidant potential of *A. barbadensis* was reduced to 58.54 to 81.10% based on HPS assay [18].

The determined antioxidant activity of *A. ferox* in ethanol, methanol, acetone and aqueous extracts by using the HPS assay, proved that the percentage inhibitions of hydrogen peroxide were dependent on solvent concentration and induced different effects, in the following order: acetone < ethanol < gallic acid < methanol < BHT (butylated hydroxytoluene) < aqueous extract [15]. Apart the solvent used for extraction it is known that there are other factors which could influence the antioxidant capacity in *Aloe* leaves such as the extraction procedure and the quantity of active compounds which exist in the parts of the plant subjected to analysis. Some authors demonstrated that the leaf skin extract exhibited the highest antioxidant activity as compared to flowers or inner parenchyma and whole leaf extracts [3, 35]. Strong correlations were established between the polyphenols and flavonoids content of leaf skin and its scavenging activity [24]. On the other hand, other researchers postulated that polysaccharides from the inner parenchyma were the main contributors to the antioxidant properties of the plant [40].

A relatively reduced radical scavenging activity of *A. barbadensis* found in this study (Fig. 6) might be associated with its lowest total carotenoid and vitamin C contents (Table 1). In a similar way, the highest antioxidant content in *A. marlothii* is reflected in an increased radical scavenging activity when compared to other species. Amongst all species, *A. spectabilis* occupy the third rank of the highest antioxidant activities, which might be ascribed also to its higher vitamin C content.

## Conclusions

The main carotenoids identified in all six *Aloe* leaves extracts studied were lutein and *β*-carotene. Therefore, *Aloe aculeata* and *Aloe ferox* species might be taken into consideration as sources of carotenoids, of which *Aloe aculeata* is the richest one in *β*-carotene and *Aloe ferox* in lutein.

According to GC–MS analysis 17 fatty acids were detected in leaves of each of *Aloe* species analysed. The most representative saturated fatty acid found in all *Aloe* species leaves was palmitic acid (C16:0), in a higher percentage being expressed in *Aloe aculeata* and *Aloe barbadensis.* As polyunsaturated fatty acids, linoleic acid (C18:2 n-6) and linolenic acid (C18:3 n-3), were better represented in *Aloe spectabilis*, *Aloe arborescens* and *Aloe ferox* leaves.

Moreover, *Aloe aculeata* has a remarkable MUFA content, by the high percentage being represented cis-7 hexadecenoic acid (C16:1 n − 7), respectively oleic acid (C18:1 n − 9). The PCA Analysis formulates a comprehensive framework encompassing the seven *Aloe* species in respect to carotenoid, fatty acid and vitamin C content. Therefore, it can be concluded that *Aloe aculeata* has a particular fatty acid content, characterised by vaccenic acid (C18:1n − 7), oleic acid (C18:1n − 9) and cis-7 hexadecenoic (C16:1n − 7) fatty acid. But, *Aloe aculeata* could be considered a rich source of carotenoids too. However, all other particular findings in other *Aloe* leaves extracts should be valued accordingly. *Aloe arborescens* and *Aloe marlothii*, followed by *Aloe ferox* and *Aloe spectabilis* proved to have the highest potential to scavenge reactive oxygen species.

Though the most literature data are mainly focused on *Aloe barbadensis*, more attention should be paid to the usage of other less-known *Aloe* species in research and industry.

## Data Availability

The data used to support the findings of this study are available from the corresponding author upon request.

## Abbreviations

DAD: Diode array detection
DPPH: 2,2-Diphenyl-1-picrylhydrazyl
GC-MS: Gas chromatography coupled with mass spectrometry
HPLC: High performance liquid chromatography
HPS: Hydrogen peroxide scavenging
ORAC: Oxygen radical absorbance capacity
FAMEs: Fatty acid methyl esters
meq: Milliequivalents
MUFA: Monounsaturated fatty acids
PUFA: Polyunsaturated fatty acids
PCA: Principle component analysis
SFA: Saturated fatty acids
TE: Trolox equivalent
TBME: Tert-butyl methyl ether
TIC: Total ion current
TPC: Total phenolic content
